# MCO Membranes: Enhanced Selectivity in High-Flux Class

**DOI:** 10.1038/srep18448

**Published:** 2015-12-16

**Authors:** Adriana Boschetti-de-Fierro, Manuel Voigt, Markus Storr, Bernd Krause

**Affiliations:** 1 Gambro Dialysatoren GmbH, Research & Development, Holger-Crafoord-Str. 26, 72379 Hechingen, Germany

## Abstract

Novel MCO high-flux membranes for hemodialysis have been developed with optimized permeability, allowing for filtration close to that of the natural kidney. A comprehensive *in vitro* characterization of the membrane properties by dextran filtration is presented. The sieving profile of pristine membranes, as well as that of membranes exposed to blood for 40 minutes, are described. The effective pore size (Stokes-Einstein radius) was estimated from filtration experiments before and after blood exposure, and results were compared to hydrodynamic radii of middle and large uremic toxins and essential proteins. The results indicate that the tailored pore sizes of the MCO membranes promote removal of large toxins while ensuring the retention of albumin.

Patients with end stage renal disease on hemodialysis have an increased mortality risk compared to the general population, which indicates that current renal replacement therapy requires improvements that translate into better patients’ outcomes. One of the unmet needs in hemodialysis is the adequate removal of uremic toxins over a broad molecular weight range. As synthetic membranes are less selective than the glomerular membrane, current hemodialysis membranes do not remove higher molecular weight toxins appropriately^1^. As a consequence, patients on hemodialysis have higher levels of middle and large molecular solutes in plasma. Additionally, hemodialysis membranes for blood purification are used generally during 4-hours-treatments, thrice weekly, while the healthy kidney operates continuously.

Membrane innovation was initially dedicated to improve biocompatibility, and is currently directed towards enhanced removal of uremic toxins and increased membrane permeability^1^,^2^. The progressive shift towards increased membrane permeability might be influenced, as mentioned, by the current gap between synthetic membranes and the natural kidney. Additionally, during the last decade some experience has been gathered with high permeability membranes such as the high cut-off membranes. Such membranes have been used in small pilot trials for controlled time periods (up to 12 weeks), and the results indicate that the expanded toxin removal might benefit the patients by decreasing the general inflammatory state^3^.

The MCO membranes were designed to deliver such expanded toxin removal as observed with the high cut-off membranes, while retaining albumin so that they are appropriate for regular use in conventional treatment schedules and treatment mode, i.e. 4-hours-treatments, thrice weekly in Europe. Here, the characterization of four experimental prototypes of the novel MCO membranes by dextran filtration is presented. Additionally, the sieving properties of the membranes before and after blood contact are reported, and the pore size during operation (i.e., hemodialysis treatment) is compared to the size of uremic toxins and vital proteins.

## Results

### Characterization of the MCO high-flux membranes by dextran sieving profiles in aqueous solution

**Figure 1:**
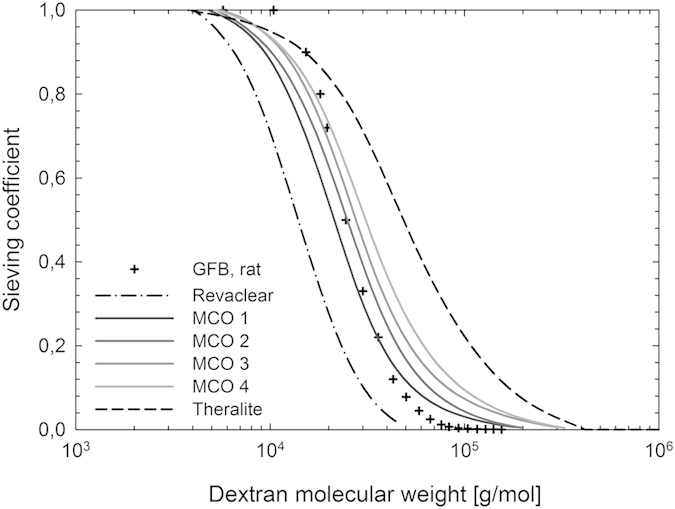
Characteristic *in vitro* dextran sieving curves measured in aqueous solution for different types of blood purification membranes: high-flux (Revaclear, MCO1-4) and high cut-off (Theralite). The data for the glomerular membrane (as reported by Axelsson *et al.*^15^) has been added for comparison (rat specimen, ficoll filtration, measured *in vivo*).

The sieving profile of the MCO high-flux membranes as determined by dextran filtration are representatively presented in Fig. 1, together with that of one conventional high-flux membranes and one high cut-off membrane. The sieving curves for the MCO membranes are located at molecular weights between that of the conventional high-flux membrane and the high cut-off membrane. The MCO sieving curves are similar to the ficoll sieving curve for the glomerular membrane.

The sieving profile of the glomerular barrier determined with ficoll is presented for the sake of comparison. The differences between dextran, ficoll and globular proteins are well documented in the literature^4^,^5^. Ficoll and dextran molecules have different molecular shapes in solution, so that the hydrodynamic radius of the molecules are different at similar molecular weight. Additionally, ficoll molecules are more symmetric, while dextrans are more flexible. In consequence, the glomerular barrier shows different selectivity towards the two types of molecules. Furthermore, the behavior of proteins in solutions and their sieving coefficient as a function of the molecular Stokes-Einstein Radius is also different. In general, the glomerular barrier is more permeable to dextrans than to ficolls, and more permeable to ficolls than to globular proteins^4^.

Table 1 depicts the values for MWRO, MWCO and pore radius (i.e., the effective Stokes-Einstein radius calculated from MWCO) for the four MCO membranes, as well as for conventional high-flux and high cut-off membranes for comparison. The differences between the MCO membranes are evident from 1 to 4, showing increased MWRO and MWCO values, which indicate increased permeability.

Furthermore, Table 2 lists the parameters describing the pore size distribution calculated from the sieving profiles for the same membranes. The presented values are average and standard deviations from the mean and variance of the log-normal distributions, assessing therefore the mean and broadness of the pore size distribution, respectively. The values of the mean of the distribution increase with membrane permeability. The variance of the respective distribution is larger as the pore sizes increase.

### Membrane characteristics after blood contact

**Figure 2:**
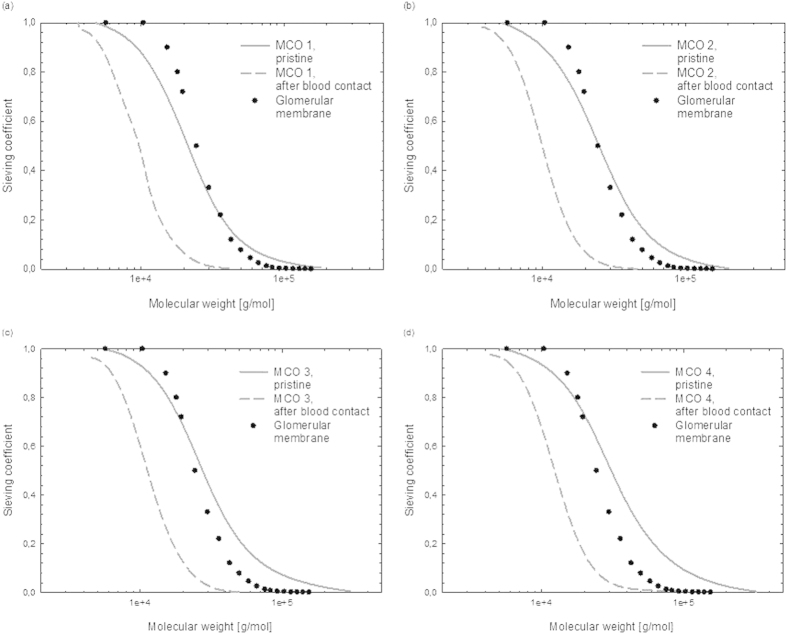
Characteristic dextran sieving curves for MCO high-flux membranes before (solid line) and after blood contact (dashed line), for (**a**) MCO 1, (**b**) MCO 2, (**c**) MCO 3 and (**d**) MCO 4. The data for the glomerular membrane (as reported by Axelsson *et al.*^15^) has been added for comparison (rat specimen, ficoll filtration, measured *in vivo*).

Figure 2 shows the sieving curves before and after exposing the MCO membranes to blood for 40 min. The untreated MCO membranes, i.e., before blood contact, are slightly more open than the glomerular membrane for molecules above 20–30 kDa. After blood exposure, the sieving curves are shifted towards lower molecular weights, and the sieving profiles indicate that the MCO membranes are less permeable than that of the glomerular membrane.

The molecular weight retention onset and molecular weight cut-off values after blood contact are also listed in Table 1 for the different MCO membranes, as well as for one conventional high-flux membrane and one high cut-off membrane. Additionally, the effective Stokes-Einstein pore radiuses calculated from those MWCO values are presented for reference. Table 2 includes the calculated pore sizes from the respective log-normal distributions after blood contact for the different membranes.

### Update on the classification of blood purification membranes

**Figure 3:**
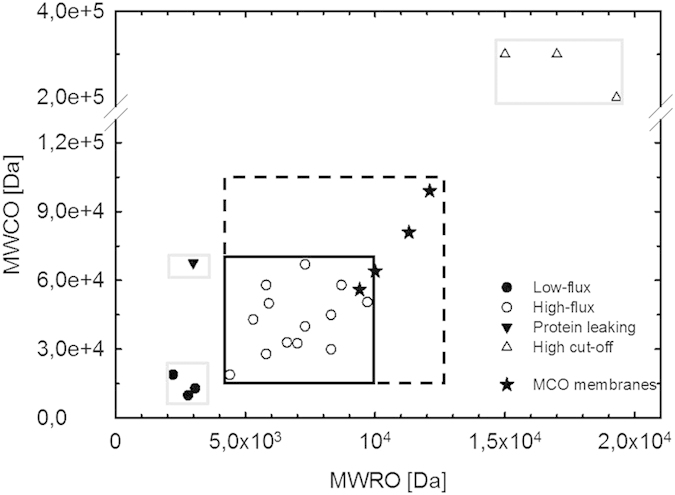
Mapping of the different types of blood purification membranes based on the molecular weight retention onset and molecular weight cut-off from dextran sieving curves. Previously published data^6^ is here completed with the values for the MCO high-flux membranes. The gray squares represent the boundaries that were included to denote low-flux, protein leaking and high cut-off membranes. The black squares enclose the high-flux dialyzers: continuous line denotes the previous classification; broken line denotes the updated classification including the MCO membranes.

We have modified the landscape of blood purification membranes previously published^6^ to include the MCO high-flux membranes (Fig. 3). The square used to denote the known high-flux dialyzers (continuous line) has been expanded to include the MWRO vs. MWCO values obtained for the MCO membranes (broken line).

## Discussion

### Characterization of the MCO high-flux membranes by dextran sieving profiles in aqueous solution

The objective of the membrane development initiative was to develop novel high-flux membranes with toxin removal capabilities similar to that of high cut-off membranes and that are also able to retain albumin adequately. The challenge resides in the membrane manufacturing process, where increasing the pore sizes usually leads to an increase in the broadness of the pore size distribution. Inevitably, the broad pore size distribution at large mean pore sizes would cause undesirable albumin permeation. Controlled membrane manufacture allows some improvement in this direction. As can be seen, the MCO 4 membrane shows similar mean pore size to that of Theralite, while having a 20% smaller variance. The less permeable versions, MCO 1 and 2, show mean pore size of around 4 nm with less than half of the variance of Theralite. This indicates that the MCO membranes offer enhanced selectivity compared to the high cut-off membranes. As expected, when compared to conventional high-flux membranes, the mean pore size and variance of the pristine membranes is larger for the MCO 1 and 2 membranes than that of Revaclear.

### Membrane characteristics after blood contact

The natural formation of the protein layer on top of the synthetic membrane during hemodialysis affects gradually the solute removal during the first 40 min of treatment. This phenomenon is illustrated by the comparison of the sieving characteristics before and after blood contact. While the pristine MCO membrane allows the passage of molecules above 70 kDa to some extent, the sieving profile of the MCO membranes (as that of every artificial membrane) shifts towards lower molecular weights during operation. This circumstance is a compromise in order to deliver a tailored removal after the inevitable membrane fouling. It has been demonstrated that the protein layer on hemodialysis membranes is completely formed after 40 min of blood contact^7^. As the hemodialysis treatment time is around 4 h in Europe, this means that during more than 80% of the treatment blood purification is accomplished by a membrane the performance of which is governed by protein fouling. The MCO membranes show sieving profiles close to that of the natural kidney after the formation of the protein layer, thereby maintaining the required performance along the treatment.

The pore size distribution for all membranes is narrower after blood contact, indicating that the selectivity of synthetic membranes improves during the operation. The correlation between pore size and broadness of the distribution is still present, although less pronounced. The advantages of the novel MCO membranes are more evident when compared to conventional high-flux membranes. For example, MCO 1 and 2 show mean pore sizes slightly larger than those for Revaclear, meanwhile their distribution variance did not increase with the mean pore size. For those membranes with larger mean pore sizes, i.e., MCO 3 and 4, the distribution variance correlates more with the pore size, increasing as the mean pore size increases.

The effective pore radius is a better indication than the mean pore size of the distribution for the biggest molecule that will pass through the membranes. The hydrodynamic radii (*R*_*h*_) for albumin and some uremic toxins are summarized in Table 3. The effective pore size of the MCO membranes is between 3.0 and 3.5 nm after the formation of the protein layer, indicating that the membranes retain albumin during treatment. Additionally, the least permeable MCO membrane has an effective pore radius of 3.0 nm during treatment, which should allow adequate removal of large uremic toxins, up to λ-FLCs.

Based on the data here presented it can be presumed that some albumin permeation takes place even after the formation of the protein layer for the most permeable membrane MCO 4. This effect, if properly controlled, is not necessarily detrimental to the patients. Hemodialysis treatment as renal replacement therapy aims to accomplish blood purification during treatment sessions (varying in time and frequency between 3 to 8 hours per session, 3 to 6 sessions per week). In contrast, a healthy kidney performs the task continuously. Therefore, renal replacement therapy is inevitably underperforming in treatment time. This fact could be partially compensated by increased removal. Albumin loss is tolerated to some extent, as demonstrated in peritoneal dialysis patients where weekly albumin losses of 21–42 g/1.73 m^2^ are accepted and not linked to outcome detriment^8^,^9^. It has also been speculated that a small and controlled albumin loss might trigger albumin synthesis in patients, which is associated with a better general state of health. For example, Krieter and Canaud have argued that since the loss of albumin was associated with the removal of, among others, advanced glycation end‐products or advanced oxidation protein products, a higher membrane permeability might even be beneficial^10^. Currently, the MCO membrane performance is being evaluated in clinical studies. Besides removal rate and clearance of middle and large uremic toxins, the albumin removal per treatment is being investigated.

### Update on the classification of blood purification membranes

The inclusion of the novel MCO membranes in the landscape of membranes for blood purification from dextran characterization evidences that these membranes expand the currently known limits of high-flux membranes. Already the MCO 1 membrane is among the best previously depicted high-flux membranes; MCO 2 expands the previous arbitrary classification beyond the currently set limits.

To deliver an appropriate renal replacement therapy is a challenge, among others, for membrane design. A blood purification membrane should mimic the filtration spectrum of the glomerular membrane, a difficult task because the glomerular membrane, as most natural membranes, shows unique selectivity and permeability. Such combination is not easily achieved with current manufacturing techniques without incorporating biological components. Additionally, the glomerular membrane suffers little to no fouling (defined as the performance detriment due to accumulation of solutes on the membrane surface) and therefore maintains its ideal performance during usage time. Renal replacement therapy is furthermore challenged by the fact that the healthy kidney accomplishes the physiological blood purification continuously Considering the limitations of synthetic membranes in renal replacement therapy, the expansion of the high-flux category is one step on the right direction that awaits confirmation in clinical application.

## Conclusions

The novel MCO membranes provide for large pore sizes with appropriate pore size distribution and permeability close to that of the natural kidney. Their MWCO values suggest that, when used in hemodialysis treatments, they allow for removal of an expanded range of uremic toxins compared to conventional high-flux membranes. Formation of a protein layer on top of the synthetic membrane during hemodialysis restricts the removal of molecules above 3.5 nm in radius, optimizing removal of large uremic toxins while ensuring retention of albumin during treatment.

## Methods

### Devices and sample preparation

Four different types of prototype devices denoted as MCO 1 to MCO 4, which differ in membrane permeability, were investigated. As reference, a high cut-off device (Theralite) and a conventional high flux membrane (Revaclear) were also tested. All devices are manufactured by Gambro Dialysatoren GmbH, Hechingen, Germany. The membrane material is a poly(aryl ether sulfone)/polyvinylpyrrolidone blend. Further device characteristics are listed in Table 4.

Membranes were characterized in minimodules, manufactured as described elsewhere^6^. All minimodules had a surface area of 360 cm^2^, nominal length of 170 mm, an effective length of approx. 120–150 mm (without PU potting) and an inner diameter of 10 mm. The internal diameter of the MCO fibers is 180 μm, and the wall thickness 35 μm, resulting in a packing density of 23.4%.

The minimodules were immersed in water for 30 min before the filtration experiments. Minimodules intended for characterization after contact with blood for simulating *in vivo* operation conditions were initially perfused with blood (bovine, 32% hematocrit, 60 g/L total protein, 16000 units/L heparin) for 40 min and rinsed afterwards with water for 30 to 60 min^7^.

### Dextran sieving coefficient test

Dextran solutions were prepared and filtration experiments were carried out as previously described^6^. For the experiments run on MCO 4, the dextran solution included one additional fraction of 150 kDa, in order to allow for sieving coefficient calculation with similar precision as for the other MCO membranes. The sieving coefficient *SC* was calculated according to equation (1) as follows:


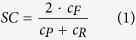


where *c*_*F*_ is the concentration of the solute in the filtrate, *c*_*P*_ its concentration in the permeate and *c*_*R*_ its concentration in the retentate.

Filtration experiments were carried out under constant shear rate (γ = 750 s^−1^) and with ultrafiltration rate set at 20% of the blood side entrance flux *Q*_*Bin*_, calculated as:


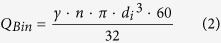


where *Q*_*Bin*_ is the flux at the blood side entrance in ml/min; *n* is the number of fibers in the minimodule; *d*_*i*_ is the inner diameter of the fibers in cm and γ is the constant shear rate mentioned above.

Experiments were carried out with n = 3, and results confidence could be increased with higher repetition number. Despite the low test repetition number, the standard deviations of the measurements remain below 10% of the value for all values but for the MWCO of Theralite. This is due to the lack of larger MW dextrans required for characterizing a membrane with such large pore sizes.

The chosen conditions assure a filtration regime since the Peclet-number for all the investigated membranes is well above 10 even for molecules in the 0.1 to 1 kDa range. Additionally, it should be noted that the filtration conditions are set to avoid backfiltration, contrary to the conditions typical of hemodialysis.

The obtained sieving curves were characterized by their molecular weight retention onset (MWRO) and molecular weight cut-off (MWCO). The MWCO is defined as the molecular weight at which the sieving coefficient is 0.1.; the MWRO describes the molecular weight at which the sieving coefficient is 0.9^6^. Since membrane pore sizes are not discrete but a distribution, we have used two different approaches from membrane technology to describe the pore size distribution: (i) the effective pore size (from the MWCO) and (ii) the mean pore size from the log-normal distribution.

(i) Pore sizes correspond to molar mass as reported by Aimar^11^ and Venturoli^4^ based on the data from Granath and Kvist^12^


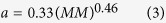


where *a* is the pore radius in Å and *MM* is the dextran molar mass in g/mol. The Stokes-Einstein radius at the MWCO corresponds to the effective membrane pore radius. The molecular weight cut-off is the molecular weight from which at least 90% of the molecules are retained by the membrane. Therefore, the hydrodynamic radius of that molecule represents the size of molecules that are retained (also at least by 90%), which is described as an effective pore size for the membranes^9^,^13^.

(ii) Pore sizes are also described by the Log-normal pore size distribution as mean pore size. Sieving curves were transformed into pore size distributions based the mentioned correlation. The distributions were evaluated as log-normal distributions and characterized by its mean and variance^14^.

## Additional Information

**How to cite this article**: Boschetti-de-Fierro, A. *et al.* MCO Membranes: Enhanced Selectivity in High-Flux Class. *Sci. Rep.* **5**, 18448; doi: 10.1038/srep18448 (2015).

## Figures and Tables

**Table 1:** Characterization of MCO hemodialysis membranes, conventional high-flux and high cut-off membranes, based on dextran sieving experiments before and after blood exposure.

	Before blood exposure			After blood exposure		
Membrane	MWRO [kDa]	MWCO [kDa]	Pore radius [nm]	MWRO [kDa]	MWCO [kDa]	Pore radius [nm]
Revaclear	5.7 ± 0.5	32 ± 3	3.9 ± 0.1	4.4 ± 0.3	14.2 ± 0.2	2.68 ± 0.02
MCO 1	9.4 ± 0.2	56 ± 3	5.0 ± 0.1	5.0 ± 0.4	18.1 ± 0.8	3.0 ± 0.1
MCO 2	10.0 ± 0.6	64 ± 3	5.4 ± 0.1	5.84 ± 0.09	18.2 ± 0.3	3.0 ± 0.1
MCO 3	11.3 ± 0.4	81 ± 9	6.0 ± 0.3	6.32 ± 0.06	22.7 ± 0.8	3.3 ± 0.1
MCO 4^a^	12.1 ± 0.7	99 ± 7	6.5 ± 0.2	6.77 ± 0.06	25 ± 5	3.5 ± 0.3
Theralite	15 ± 1	300 ± 100	10 ± 2	8.1 ± 0.8	40 ± 8	4.3 ± 0.4

Values are average ± standard deviation for *n* = 3.

^a^Experiments for MCO 4 included also one dextran fraction of 150 kDa.

**Table 2:** Mean (pore radius) and variance of the log-normal pore size distribution for the four MCO prototype membranes, conventional high-flux and high cut-off membranes before and after blood exposure.

Membrane	Before blood exposure		After blood exposure	
	Mean [nm]	Variance [nm]	Mean [nm]	Variance [nm]
Revaclear	3.0 ± 0.3	2 ± 1	2.42 ± 0.08	0.587 ± 0.003
MCO 1	4.1 ± 0.2	4.7 ± 0.8	2.5 ± 0.1	0.6 ± 0.2
MCO 2	4.0 ± 0.2	4.6 ± 0.4	2.55 ± 0.07	0.7 ± 0.1
MCO 3	4.40 ± 0.03	6.4 ± 0.1	2.9 ± 0.1	1.3 ± 0.4
MCO 4	4.8 ± 0.2	8.8 ± 0.4	3.3 ± 0.6	3 ± 3
Theralite	5.1 ± 0.3	11 ± 2	3.4 ± 0.2	2 ± 1

Values are average ± standard deviation for *n* = 3.

**Table 3:** Hydrodynamic radius (*R*_*h*_) for albumin and some representative middle and large uremic toxins.

Molecule	*R*_*h*_ [nm]	Comments	Ref.	
β_2_ microglobulin	1.7	calculated from the diffusion coefficient in free solution	16	
Tumor necrosis factor (TNFα)	1.9 − 2.3	depending on its aggregation state, influenced by concentration and pH	17	
Free light chains (FLC) monomeric state (mostly κ-FLC)	2.3	Stokes’ radius determined by chromatography	18	
Free light chains (FLC) dimeric form (mostly λ-FLC)	2.8	Stokes’ radius determined by chromatography	18	
Albumin	3.51	calculated from the intrinsic viscosity (agrees with Stokes’ radii from diffusion and sedimentation coefficient)	19	

**Table 4:** Characteristics of the devices used for membrane characterization with dextran filtration.

Device	Inner diameter /wall thickness [μm]	Surface area [m^2^]	UFC [mL/h*mmHg]	Membrane Type
Revaclear	190/35	1.8	60	High-flux
MCO 1	180/35	1.7	48	High-flux
MCO 2	180/35	1.7	52	High-flux
MCO 3	180/35	1.7	49	High-flux
MCO 4	180/35	1.7	50	High-flux
Theralite	215/50	2.1	52	High Cut-off
